# The assessment of quality of life in acute cough with the Leicester Cough Questionnaire (LCQ-acute)

**DOI:** 10.1186/1745-9974-7-4

**Published:** 2011-07-18

**Authors:** Nadia Yousaf, Kai K Lee, Bhagyashree Jayaraman, Ian D Pavord, Surinder S Birring

**Affiliations:** 1 Institute for lung health, Department of Respiratory medicine, Glenfield Hospital, Leicester, UK; 2 King's College London, Division of Asthma, Allergy and Lung Biology, London, UK

## Abstract

**Introduction:**

Acute cough has a significant impact on physical and psychosocial health and is associated with an impaired quality of life (QOL). The Leicester Cough Questionnaire (LCQ) is a validated cough-related health status questionnaire designed for patients with chronic cough. The purpose of this study was to validate the LCQ for the assessment of health related QOL in patients with acute cough and determine the clinical minimal important difference (MID).

**Methods:**

10 subjects with cough due to acute upper respiratory tract infection underwent focused interviews to investigate the face validity of the LCQ. The LCQ was also evaluated by a multidisciplinary team. 30 subjects completed the revised LCQ-acute and a cough visual analogue score (VAS: 0-100 mm) within one week of onset of cough and again <2 weeks later and at resolution of cough. The concurrent validity, internal reliability, repeatability and responsiveness of the LCQ-acute were also assessed. Patients also completed a Global Rating of Change Questionnaire that assessed the change in cough severity between visits. The MID was calculated as the change in LCQ-acute score for patients responding to GRCQ category representing the smallest change in health status that patients found worthwhile.

**Results:**

Health status was severely impaired at baseline affecting all domains; median (interquartile range) total LCQ-acute score 13.0 (3.4). All subjects found the LCQ-acute questionnaire acceptable for assessing their cough. Internal reliability of the LCQ-acute was good for all domains and total score, Cronbach's α coefficients >0.9. There was a significant correlation between LCQ-acute and VAS (ρ = -0.48, p = 0.007). The LCQ-acute and its domains were highly responsive to change; effect sizes 1.7-2.3. The MID for total LCQ and VAS were 2.5 and 13 mm respectively.

**Conclusion:**

The LCQ-acute is a brief, simple and valid instrument to assess cough specific health related QOL in patients with acute cough. It is a highly responsive tool suggesting that it will be particularly useful to assess the effect of antitussive therapy.

## Introduction

Acute cough impacts significantly on physical and psychosocial health, leading to impairment in quality of life (QOL) [1]. Chest pain, nausea and sleep disturbance are particularly common [2]. Twenty million work days are lost each year in the USA due to acute cough according to the National Centre for Health Statistics [3]. The assessment of cough severity in acute cough is limited to self reported symptom scales, scores or diaries. There is increasing recognition that health related quality of life assessment is important, particularly in the evaluation of therapy. We have previously reported the development and validation of the Leicester Cough Questionnaire (LCQ) which is a brief, self completed, widely used, health related QOL questionnaire for chronic cough [4]. It is not known if the LCQ could be used to assess QOL in acute cough. The aim of this study was to adapt, validate and assess the LCQ for patients with acute cough and to determine the minimal important difference (MID).

## Methods

### Subjects

30 subjects (10 men) with cough due to acute upper respiratory tract infection were recruited within one week of onset of symptoms. Patients were recruited during the peak cough/cold season October to April. An upper respiratory tract infection was considered a cause of acute cough if subjects had 2 or more symptoms at least 1 day prior to the study of: rhinorrhoea, sneezing, fever, myalgia, malaise, headache and sore throat [5]. Subjects with a history of respiratory disease, chronic cough or those taking antitussive or upper respiratory tract infection drugs or angiotensin converting enzyme inhibitors were excluded. 1 patient had a history of seasonal allergic rhinitis. Informed consent was obtained from all patients and the study was approved by the local research ethics committee.

### Questionnaires

#### Leicester Cough Questionnaire (LCQ)

The LCQ is a 19 item questionnaire that assesses cough-related QOL [4]. It has 3 domains (physical, psychological and social). The total score range is 3-21 and domain scores range from 1-7; a higher score indicates a better quality of life. The questionnaire was revised so that each item related to the patient's experience within a 24 hour time frame (see Additional File 1).

#### Cough Visual Analogue Scale (VAS)

The cough VAS is a 100 mm scale on which patients indicate the severity of cough [6].

#### Global Rating of Change Questionnaire (GRCQ)

The GRCQ is a 15 point scale widely used to determine the MID of health related QOL questionnaires [7]. Patients were asked to rate global changes in health and sub-domains using 4 GRCQs. The GRCQ response ranged from -7 (a great deal worse) to +7 (a great deal better) and was classified as unchanged (-1,0,+1), small change (-3,-2,+3,+2), moderate change (-5, -4, +5, +4) and large change (-7, -6, +7, +6). MID was defined as the change in LCQ score corresponding to a small change in GRCQ score.

### Protocol

The LCQ and VAS were completed on three occasions. Patients completed the LCQ-1, VAS-1 and a structured questionnaire designed to record demographics and symptoms associated with acute cough within one week of onset. Patients were asked to complete a GRCQ and a repeat LCQ-2 and VAS-2 within 2 weeks of LCQ-1 and again when the cough resolved (LCQ-3 and VAS-3.)

### Validation

#### 1. Face Validity

The suitability of the wording and content of the LCQ for detecting health related QOL in patients with acute cough was assessed by:

a. A literature review of QOL assessment in acute cough.

b. Review of the LCQ by a multidisciplinary team (doctor, nurse, physiotherapist, pharmacist)

c. Focussed interviews with 10 patients with acute cough to assess its impact on QOL and to ascertain their views on the suitability of the LCQ to assess QOL.

#### 2. Concurrent Validity

Concurrent validity is the assessment of an instrument against other standards; it was assessed by correlating LCQ-1 scores with cough VAS-1.

#### 3. Internal Reliability

Internal reliability of each domain was assessed by determining Cronbach's alpha coefficients which indicate the extent to which items are related. Internal reliability is generally acceptable if Cronbach's alpha coefficient is greater than 0.7.

#### 4. Repeatability

The repeatability of the LCQ was assessed in those patients indicating no change in health status on the GRCQ over 2 weeks.

#### 5. Responsiveness

The responsiveness of the LCQ and VAS was determined by calculating the effect size of change between baseline and resolution of the cough.

#### 6. Minimal Important Difference

The MID of the LCQ and VAS were determined using anchor based methods using the GRCQ as described by Juniper [7].

### Statistical Analysis

SPSS version 16 was used for data analysis. Data are presented as mean (standard error of the mean or standard deviation) or median (inter-quartile range) according to its distribution. In accordance with previous studies we expressed global rating scores as absolute numbers i.e. when the change was negative, the sign was reversed as was the sign of change in LCQ score [8]. Spearman's correlation coefficient was used to determine concurrent validity. Mann Whitney tests were used to compare groups. Internal reliability was tested by determining Cronbach's alpha coefficient. Repeatability was assessed by determining the intra class correlation coefficients.

## Results

All patients that were interviewed found the LCQ suitable for use in acute cough. The only modification to the LCQ after review by the multidisciplinary meeting was alteration of the time frame for each item from 2 weeks to the past 24 hours. See Additional File 1 for the final version of LCQ-acute. 2 patients did not complete the GRCQ and their data was excluded from the validation of the MID. Subject characteristics are given in table 1. Health related QOL was impaired at baseline; median (IQR) total LCQ score 12.8 (3.4), physical 4.5 (1.1), psychological 4.9 (1.1) and social 4 (1.4). There were no significant gender differences in VAS, LCQ or GRCQ scores.

**Table 1:** Subject characteristics (n = 30)

Characteristic	
Age mean (SD)	32 (10)
Male n (%)	10 (33)
Smokers n (%)	2 (7)
Non smokers n (%)	28(93)
Duration of cough in days (SD)	12 (9)
LCQ score baseline median(IQR) all patients	12.8 (14.9; 11.5)
LCQ score baseline median (IQR) females	13.5 (15.8; 11.2)
LCQ score baseline median (IQR) males	13.4 (16.5; 10.3)
VAS score baseline mean(SD)mm all patients	39 (25)
VAS score baseline mean(SD)mm females	39 (26)
VAS score baseline mean(SD)mm males	37 (23)
Tiredness n (%)	24 (80%)
Sore throat	18 (60)
Runny nose	17 (57)
Sneezing	16 (53)
Headache	16 (53)
Clear sputum	14 (47)
Coloured sputum	14 (47)
Aches/pains	12 (40)
Fever	10 (33)
Facial pain	9 (30)

**Figure 1:**
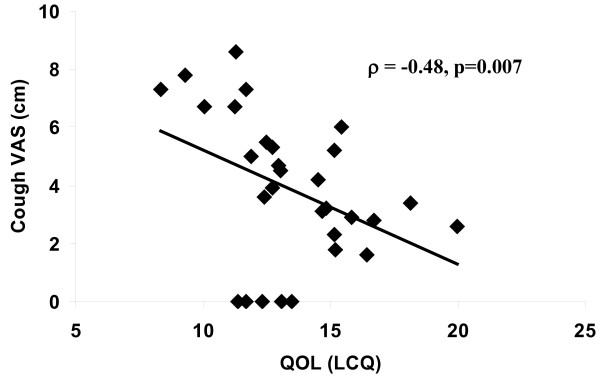


There was a significant correlation between the cough VAS and the LCQ total score at baseline (ρ = -0.48, p = 0.007; figure 1). Internal consistency was high for all domains and total LCQ score (table 2). Only 4 patients indicated a GRCQ score of 0, 2 patients indicated a GRCQ score of 1; this sample size was considered too small to determine intraclass coefficient of repeatability.

**Table 2:** Internal consistency reliability (Cronbach's Alpha coefficients)

LCQ	Cronbach's Alpha Coefficient
Total	0.94
Social	0.90
Psychological	0.90
Physical	0.95

QOL improved between visits 1 and 2; median LCQ score 12.8 vs 16.7; p <0.001. QOL improved in all but one patient between visits 1 and 2. The median change in LCQ score for each GRCQ category is given in table 3. The LCQ MID corresponding to a small change in the GRCQ was 2.5 (table 3). The correlation between GRCQ score and change in LCQ total was r = 0.6 (p = 0.001) and for domains: physical r = 0.51 (p = 0.05), psychological r = 0.46 (p = 0.02) and social r = 0.47 (p = 0.01). The LCQ and VAS were responsive to reductions in cough severity (table 4). There was a weak relationship between change in VAS score and change in LCQ score (r = 0.37, p = 0.05). The MID for VAS was 13 mm. There was no correlation between change in VAS and GRCQ score (r = 0.02, p = 0.78).

**Table 3:** Change in Leicester cough questionnaire score and visual analogue score per global rating of change category

	Global rating of change questionnaire categories							
	Unchanged	(-1/0/1)	Small	(-3/-2/2/3)	Moderate	(-5/-4/4/5)	Large	(-7/-6/6/7)
**Change in LCQ total score**	N = 6	1.2 (0.9)	N = 12	2.5 (3.1)	N = 6	4.6 (2.9)	N = 4	6.8 (3.5)
**Change in LCQ physical score**	N = 1	(0.6)	N = 14	0.6 (0.8)	N = 8	1.0 (0.8)	N = 5	1.9 (1.5)
**Change in LCQ psychological score**	N = 9	0.1 (1.0)	N = 8	0.7 (1.2)	N = 7	1.4 (0.9)	N = 4	2.2 (1.5)
**Change in LCQ social score**	N = 6	0.6 (0.4)	N = 14	0.9(1.4)	N = 5	2.3 (0.3)	N = 3	2.5 (0.6)
Change in cough VAS score (mm) *	N = 6	7.0 (0.6)	N = 12	13.0 (0.6)	N = 6	13.0 (0.6)	N = 4	33.0 (2.3)

N = number of cases. Median (interquartile range) except * mean (standard deviation).

**Table 4:** Responsiveness of LCQ-acute: Effect sizes

	Effect size
	
LCQ Total	2.3
LCQ Social	1.7
LCQ Psychological	1.8
LCQ Physical	2.3
	
VAS	1.4

## Discussion

The LCQ-acute is a valid health status measure for patients with acute cough. It is easy to use, self administered and takes less than 5 minutes to complete. The LCQ-acute was highly responsive to change, suggesting it might be particularly useful in assessing the response to treatment both in clinic and in clinical trials. The minimal important difference, the smallest change in health status patients find worthwhile was a change in LCQ-acute score of 2.5.

We validated the LCQ-acute for acute cough using a well accepted QOL instrument development methodology [9]. The only alteration to the original LCQ was a reduction in the assessment period from 2 weeks to 24 hours to reflect the rapid change in symptoms associated with acute cough. The validity of the LCQ-acute was comparable to the original LCQ used by patients with chronic cough; face and concurrent validity, internal reliability and responsiveness were within acceptable standards for quality of life questionnaires [9]. We were unable to determine the repeatability of the LCQ-acute since most patients reported improvement in cough severity within the time frame of this study. A shorter time interval between test and retest questionnaires or a much larger study may allow the determination of repeatability coefficients in future. It is possible that symptoms of upper respiratory tract infection other than cough may have influenced quality of life. The LCQ-acute questionnaire items were however individually phrased to be relevant to cough.

The MID for LCQ-acute was 2.5. This should facilitate the interpretation of health status data from clinical studies and calculate sample sizes for future studies. The MID was greater than that for patients with chronic cough (1.3) [8]. This may be due to small changes in quality of life having a larger impact in chronic conditions due to the cumulative effect of living with the symptom for many years. We chose anchor based methodology to determine the MID rather than distribution methods based on standard deviations since the latter depend on the heterogeneity of the population under study and utilises arbitrary units of measure [10-12]. There are limitations with the anchor based methodology. We included patients with GRCQ scores +/- 1 in the "unchanged" category and it is therefore possible that some patients may have experienced a significant change in cough. We chose this method to be consistent with those described by Juniper; [7] moreover, they have previously reported that a GRCQ score of +/- 1 does not represent clinically significant change. The GRCQ is a subjective instrument and subject to recall bias. Our findings need confirmation with objective assessment of cough severity such as cough reflex sensitivity measurement and cough monitoring. The time-frame for GRCQ was relatively short and this may have minimised the effect of recall bias. The determination of the MID by prospective methodology avoids some of the limitations of the anchor based methods; this deserves consideration in future studies (Irwin RS, personal communication and data in press). We found a significant correlation between GRCQ and the change in LCQ-acute scores supporting the use of the GRCQ. There was a step-wise increase in change in LCQ-acute scores across GRCQ categories, which suggests that LCQ-acute can discriminate patients with small and large changes in health status. Our study demonstrates that health status improves in the vast majority of patients with acute cough. Further studies will be needed to determine if a MID of 2.5 is applicable for patients whose health status deteriorates.

We were unable to perform a subanalysis to determine whether the MID varied according to age, gender or strain of virus; this will require further investigation. We determined the LCQ-acute MID in a natural recovery study design. It may be difficult to establish the MID in patients taking currently available antitussive drugs since the relative improvement in cough severity due to natural recovery, placebo effect and therapeutic effect of the antitussive drug are not clear. We suggest that antitussive drugs should aim to achieve a clinical benefit that is greater than an increase of LCQ-acute score of at least 2.5 units. This should ideally be achieved at an earlier phase of the illness.

The impairment in quality of life suffered by our cohort of subjects with acute cough was comparable to that of chronic cough [13]. The impairment in QOL was moderate to severe but transient compared to chronic cough. All health domains were affected. A significant impairment in the health status of patients with acute cough was also found in a study using the CQLQ, another validated cough specific health status questionnaire for patients with acute and chronic cough [1]. Although this seems surprising for such a common and benign condition, it reflects the fact that the LCQ-acute and CQLQ are cough specific health measures. It is likely that general health related QOL determined by generic tools such as the SF36 will demonstrate a lesser impact on QOL in acute compared with chronic cough.

This is the first study to validate the cough VAS in subjects with acute cough and determine its MID. The VAS is easier to use and widely recognised compared to QOL tools. QOL tools however have the advantage that they quantify overall health status and identify the subdomains of health affected. The relationship between VAS and QOL was less strong than that for patients with chronic cough and there was no relationship between the global health assessment tools (GRCQ) and VAS in contrast to the LCQ-acute. This suggests that VAS cannot be used as a substitute for health related QOL tools. Furthermore, we have demonstrated that the LCQ-acute is more responsive to changes in cough severity than the VAS.

In conclusion, there are a range of options available to assess cough severity in acute cough. The LCQ-acute should be used to complement other subjective tools and objective tools such as cough reflex sensitivity and ambulatory cough frequency monitoring. The LCQ-acute represents an advance in the assessment of cough severity and should aid clinicians and researchers in making meaningful interpretations of health related QOL outcomes.

## Funding

Departmental funding

## Conflict of interest statement

None of the authors has a financial relationship with a commercial entity that has an interest in the subject of this manuscript.

## Authors' contributions

NY: Data collection, analysis of results, wrote the manuscript

KKL: Data analysis and review of manuscript

BJ: data collection and analysis.

IDP: Reviewed the manuscript.

SSB: Designed study, analysis and reviewed manuscript

All authors have read and approved the final manuscript.

## Supplementary Material

Additional file 1 **Concurrent validity: relationship between QOL and cough VAS**. This figure shows an inverse significant correlation between cough VAS and QOL as measured by the LCQ. QOL: quality of life, VAS: visual analogue scale, LCQ: Leicester Cough Questionnaire. Click here for file
