# Supplementary material for: The assessment of quality of life in acute cough with the Leicester Cough Questionnaire (LCQ-acute)
Source: Cough. 2011 Jul 18;7:4. doi: 10.1186/1745-9974-7-4 (PMC3169450; doi:10.1186/1745-9974-7-4)
Supplement: Additional file 1 — Concurrent validity: relationship between QOL and cough VAS. This figure shows an inverse significant correlation between cough VAS and QOL as measured by the LCQ. QOL: quality of life, VAS: visual analogue scale, LCQ: Leicester Cough Questionnaire. [file 1745-9974-7-4-S1.DOC]

**APPENDIX : LEICESTER COUGH QUESTIONNAIRE-acute (LCQ-acute)**

This questionnaire is designed to assess the impact of cough on various aspects of your life. Read each question carefully and answer by CIRCLING the response that best applies to you. Please answer ALL questions, as honestly as you can.

**1.** In the last 24-hours, have you had chest or stomach pains as a result of your cough?

1 2 3 4 5 6 7

All of the time Most of the time A good bit of the time Some of the time A little of the time Hardly any of the time None of the time

**2.** In the last 24-hours, have you been bothered by sputum (phlegm) production when you cough?

1 2 3 4 5 6 7

Every time Most times Several times Some times Occasionally Rarely Never

**3.** In the last 24-hours, have you been tired because of your cough?

1 2 3 4 5 6 7

All of the time Most of the time A good bit of the time Some of the time A little of the time Hardly any of the time None of the time

**4.** In the last 24-hours, have you felt in control of your cough?

1 2 3 4 5 6 7

None of the time Hardly any of the time A little of the time Some of the time A good bit of the time Most of the time All of the time

**5.** How often during the last 24-hours have you felt embarrassed by your coughing?

1 2 3 4 5 6 7

All of the time Most of the time A good bit of the time Some of the time A little of the time Hardly any of the time None of the time

**6.** In the last 24-hours, my cough has made me feel anxious

1 2 3 4 5 6 7

All of the time Most of the time A good bit of the time Some of the time A little of the time Hardly any of the time None of the time

**7.** In the last 24-hours, my cough has interfered with my job, or other daily tasks

1 2 3 4 5 6 7

All of the time Most of the time A good bit of the time Some of the time A little of the time Hardly any of the time None of the time

**8.** In the last 24-hours, I felt that my cough interfered with the overall enjoyment of my life

1 2 3 4 5 6 7

All of the time Most of the time A good bit of the time Some of the time A little of the time Hardly any of the time None of the time

**9.** In the last 2 weeks, exposure to paints or fumes has made me cough

1 2 3 4 5 6 7

All of the time Most of the time A good bit of the time Some of the time A little of the time Hardly any of the time None of the time

**10.** In the last 24-hours, has your cough disturbed your sleep?

1 2 3 4 5 6 7

All of the time Most of the time A good bit of the time Some of the time A little of the time Hardly any of the time None of the time

**11.** In the last 24-hours, how many times have you had coughing bouts?

1 2 3 4 5 6 7

All the time (continuously) Most times of during the day Several times during the day Some times during the day Occasionally through the day Rarely None

**12.** In the last 24-hours, my cough has made me feel frustrated

1 2 3 4 5 6 7

All of the time Most of the time A good bit of the time Some of the time A little of the time Hardly any of the time None of the time

**13.** In the last 24-hours, my cough has made me feel fed up

1 2 3 4 5 6 7

All of the time Most of the time A good bit of the time Some of the time A little of the time Hardly any of the time None of the time

**14.** In the last 24-hours, have you suffered from a hoarse voice as a result of your cough?

1 2 3 4 5 6 7

All of the time Most of the time A good bit of the time Some of the time A little of the time Hardly any of the time None of the time

**15.** In the last 24-hours, have you had a lot of energy?

1 2 3 4 5 6 7

None of the time Hardly any of the time A little of the time Some of the time A good bit of the time Most of the time All of the time

**16.** In the last 24-hours, have you worried that your cough may indicate a serious illness?

1 2 3 4 5 6 7

All of the time Most of the time A good bit of the time Some of the time A little of the time Hardly any of the time None of the time

**17.** In the last 24-hours, have you been concerned that other people think something is wrong with you, because of your cough?

1 2 3 4 5 6 7

All of the time Most of the time A good bit of the time Some of the time A little of the time Hardly any of the time None of the time

**18.** In the last 24-hours, my cough has interrupted conversation or telephone calls

1 2 3 4 5 6 7

All of the time Most of the time A good bit of the time Some of the time A little of the time Hardly any of the time None of the time

**19.** In the last 24-hours, I feel that my cough has annoyed my partner, family or friends

1 2 3 4 5 6 7

Every time I cough Most times when I cough Several times when I cough Some times when I cough Occasionally when I cough Rarely Never

Thank you for completing this questionnaire.

**LCQ Scoring**

1. Domains (questions): Physical: 1,2,3,9,10,11,14,15

Psychological 4,5,6,12,13,16,17

Social: 7,8,18,19

2. Domain Scores: total score from items in domain / number of items in domain (range 1-7)

3. Total Scores: Addition of domain scores (range 3-21)
